# The network of sheep movements within Great Britain: network properties and their implications for infectious disease spread

**DOI:** 10.1098/rsif.2006.0129

**Published:** 2006-04-28

**Authors:** Istvan Z Kiss, Darren M Green, Rowland R Kao

**Affiliations:** Department of Zoology, University of OxfordSouth Parks Road, Oxford OX1 3PS, UK

**Keywords:** epidemiology, disease spread, sheep movements, networks, mixing, small-world

## Abstract

During the 2001 foot and mouth disease epidemic in the UK, initial dissemination of the disease to widespread geographical regions was attributed to livestock movement, especially of sheep. In response, recording schemes to provide accurate data describing the movement of large livestock in Great Britain (GB) were introduced. Using these data, we reconstruct directed contact networks within the sheep industry and identify key epidemiological properties of these networks. There is clear seasonality in sheep movements, with a peak of intense activity in August and September and an associated high risk of a large epidemic. The high correlation between the in and out degree of nodes favours disease transmission. However, the contact networks were largely dissasortative: highly connected nodes mostly connect to nodes with few contacts, effectively slowing the spread of disease. This is a result of bipartite-like network properties, with most links occurring between highly active markets and less active farms. When comparing sheep movement networks (SMNs) to randomly generated networks with the same number of nodes and node degrees, despite structural differences (such as disassortativity and higher frequency of even path lengths in the SMNs), the characteristic path lengths within the SMNs are close to values computed from the corresponding random networks, showing that SMNs have ‘small-world’-like properties. Using the network properties, we show that targeted biosecurity or surveillance at highly connected nodes would be highly effective in preventing a large and widespread epidemic.

## 1. Introduction

In the 2001 foot and mouth disease (FMD) epidemic in the UK, livestock movements, especially of sheep, caused the initial dissemination of FMD to different parts of the UK (Gibbens *et al*. 2001; Kao 2002). This has prompted the recording of livestock movements to aid disease surveillance and control within the livestock industry (Bourn 2003). Sheep are not particularly susceptible to FMD; however once infected, it is difficult to identify clinical signs (Davies 2002). Therefore, they may spread disease undetected, as occurred in 2001 (Gibbens *et al*. 2001). Understanding the structure of the sheep industry, therefore, is important for preventing and controlling future epidemic outbreaks.

The extensive detail of the livestock movement dataset makes it well suited for the use of methodologies developed within graph theory and social network analysis. The contact network structure has important implications for disease invasion and spread (Anderson & May 1991; Liljeros *et al*. 2001; May & Lloyd 2001; Pastor-Satorras & Vespignani 2001; Hufnagel *et al*. 2004; Meyers *et al*. 2005), and its study can provide scientific support for the development and implementation of effective preventive and control measures.

Kao *et al*. (2006) have recently analysed the dynamic livestock movement network of Great Britain using a simple methodology where a network of epidemiological contacts is derived from all the potentially infectious movements. They related the percolation of the disease through this ‘epidemiological network’ to the basic reproduction ratio *R*_0_. However, their approach is largely data-driven and it remains useful to understand the livestock network in the context of existing network theory. As a prelude to more analytical studies, we identify key epidemiological characteristics of the highly diverse sheep livestock network (Pollott 1998) that distinguish it from baseline networks with randomly distributed connections. The latter form the basis of most prior studies of disease transmission on networks, and so we discuss possible consequences for disease spread and control.

Using parameters appropriate for FMD, an individual-based SEI model is used to simulate the spread of the disease on the sheep movement network (SMN). The 2001 FMD epidemic in the UK involved several livestock species and movements were not the only pathways for disease spread. However, movement of sheep carrying undetected disease was largely responsible for most of the initial dissemination of FMD around the UK and provided the infectious seeds for virtually all localized regional epidemics (Gibbens *et al*. 2001; Kao 2002). The delay between the appearance of an infectious agent and its detection provides a time window when disease can spread via livestock movements and our analysis addresses this initial stage of disease spread.

Using this model, results and predictions from the network theory approach are tested. Results from epidemics spreading on the SMN are compared to epidemics propagating on randomly generated networks, with the same number of nodes and same in and out degrees as the SMN. Finally, the effectiveness of targeted removal is explored by modelling the removal of highly active nodes.

## 2. Material and methods

### 2.1 Network construction

Sheep movements are recorded on the Animal Movements Licensing System (AMLS) and Scottish Animal Movement System (SAMS) databases maintained and administered by Department for Environment, Food and Rural Affairs (DEFRA) and Scottish Executive Environment and Rural Affairs Department (SEERAD), respectively. These databases contain the date, source, destination, species type and batch size of the movements of large livestock. Both systems have been in operation since the beginning of 2002, but full implementation of the system was not immediately achieved. Therefore, data prior to 2003 are excluded in the analysis below. This study concentrates on data prior to 30 November 2004, at which time changes in the data recording system were implemented, also matching the timeframe analysed in Kao *et al*. (2006).

Based on these livestock movement databases, directed networks of sheep movements can be reconstructed. Each node represents a livestock holding listed as source or destination in the movement databases. The directed links between nodes represent livestock movements. Consistent with the 2001 epidemic (Gibbens *et al*. 2001) the network of sheep movements is broken down in consecutive four-week periods, beyond which it is assumed unlikely that an epidemic could persist without being identified.

In order to create a static network for analysis, any pair of nodes is considered connected by a directed link if, during a single four-week period, there is at least one movement of sheep between them. The constructed networks are static, containing all the movements that happened within a four-week period irrespective of their relative timings. Markets in the database are identified from the national June Agricultural Census (2003). Regulations require that all livestock be moved from a market within 48 h of arrival. This emptying of markets between trading days and disinfection of market premises minimizes transmission between livestock present on the markets on different trading days. Thus, each market is considered to be a different node on each day that movement to or from a market occurs, though in practice this is a lower limit. For example, a real market labelled A is represented by two distinct nodes on day D_1_ and on day D_2_. Market A with all the on and off moves that occur on day D_1_ is represented by a node that is different from the node that represents market A with all the on an off moves that occur on day D_2_.

### 2.2 Network properties

In a directed contact network, a crucial role in disease transmission is played by the strong components (Newman *et al*. 2001). These are defined as subsets of the network where any two nodes *i* and *j* are mutually reachable by following directed paths, and thus a disease introduced into any node in a strong component can potentially reach any other node in that strong component. The largest strong component is known as the giant strongly connected component (GSCC). Using Tarjan's algorithm (Sedgewick 2002), the strong components were determined for each consecutive four-week period from 1 January 2003 until 30 November 2004. Within the GSCCs, the distribution of contacts, clustering, the correlation between the in and out degree of the nodes and the correlation between the degrees of connected nodes was examined in detail.

Dijkstra's algorithm (Sedgewick 2002) was used to compute the minimal path lengths between all possible pairs of nodes (i.e. the minimal number of links that are needed to connect two nodes) within the SMNs. The average path length, the distribution of path lengths and the diameter of the SMNs (i.e. the longest minimal path length) provide information about the possibility of accessing nodes through the network. The shorter the path length between two nodes, the more likely one node is to become infected, should the other already be infected. A shorter diameter means that the number of generations for a disease to spread throughout the SMN is reduced.

### 2.3 Network epidemic simulations

To understand the effect of network properties on the spread of disease and to evaluate the extent of a potential epidemic outbreak prior to the discovery of disease, epidemic simulations on the SMNs and theoretical random networks were compared, using an individual-based SEI model. Theoretical random networks were generated using the same number of nodes and the same in and out degrees for each node as found in the SMNs. However, the links between the nodes were placed at random using the configuration model (Bollobás 1980). There are virtually no degree correlations of connected nodes present in these random networks: links were placed independently of the degree of source and destination nodes.

The epidemic simulations focus on the initial spread of FMD. In the 2001 FMD epidemic in the UK, the disease remained and spread undetected for a period of approximately 28 days (Gibbens *et al*. 2001). During this time window, the movement of livestock was not banned and no epidemic control measures were in place. To our knowledge, although there is some experimental evidence that multiple cycles of transmission in sheep leads to decreasing viraemia (Hughes *et al*. 2002), there is no estimate for the flock level infectious period in the 2001 epidemic. Hence, we make the worst-case assumption that infectious premises stayed infectious until the presence of the disease was discovered and control measures were put in place. After the presence of the disease was discovered, a movement ban was imposed and infectious and potentially infectious farms were targeted for control. Therefore, to model the initial spread through movements of sheep, an SEI model is used, where ‘S’ represents susceptible nodes and ‘E’ and ‘I’ represent exposed and infectious nodes, respectively. Recovery is not considered here, since in the time-period of interest, the disease was spreading undetected and no control measures were in place. To account for the movement ban, simulations are limited to 28 days. The probability *p* of a susceptible node with *k* infectious neighbours becoming exposed in a small interval of time Δ*t* is given by p=1−exp(τkΔt). Here, *τ* is the probability per unit time of infection spreading through a single contact between an infectious and a susceptible node. An exposed node becomes infectious at rate *δ*, with the duration of the latency period of 1/*δ*=3 days (Gibbens *et al*. 2001). For the purpose of the simulation *τ* was varied. The epidemics were seeded with ten randomly chosen nodes to avoid early stochastic extinction. Results were averaged over 100 different network realizations and 100 epidemic realizations on each network.

## 3. Results

### 3.1 Network properties

**Figure 1:**
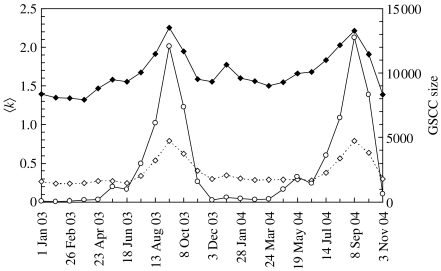
The average number of connections per node 〈*k*〉 and the size of the giant strongly connected component (GSCC) for networks built by considering consecutive four-week periods starting on 1 January 2003 until 30 November 2004. The continuous line (solid diamonds) represents the average considering only those nodes that were active during the considered four-week period. The dotted line (open diamonds) represents the average considering all the nodes that were involved in movements during the whole period of study. The continuous line (open circles) represents the GSCC size.

Over the period studied, 131 927 different nodes were identified as sources and destinations for sheep movements. In figure 1, the average number of connections per node (〈*k*〉) is plotted for each four-week period. There is a strong seasonal effect, with a maximum in the number of movements in August and September of each year. This increased activity suggests that during this period the livestock network is particularly vulnerable to large epidemics.

**Figure 2:**
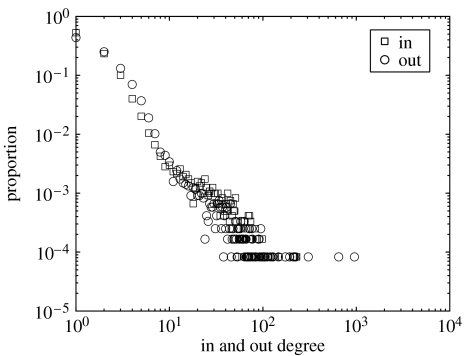
The in and out degree distribution of the sheep movement network starting on 8 September 2004.

The in and out degree distributions of a single network, representing the four-week period starting on 8 September 2004, are presented in figure 2. This period was chosen to give a well-connected network, when the SMN is expected to be most vulnerable to an epidemic. Both in and out degree distributions within this network show scale-free properties with high heterogeneity in the number of contacts per node. Similar qualitative behaviour is observed for SMNs generated over different time frames. Markets in general tend to have a higher number of in and out links compared to farms, even when separated into unique ‘market-days’ (not shown).

**Figure 3:**
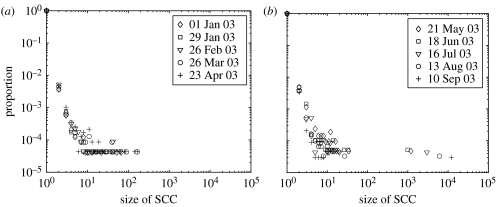
The size distribution of the strongly connected components. (*a*) Below the percolation threshold, the network is fragmented in components of small sizes. (*b*) Above the percolation threshold, the GSCC becomes isolated from the remaining components. Distributions over the different time-periods considered are consistent. The isolated points on the right represent the GSCCs.

For each four-week period, the strong components were identified using Tarjan's algorithm. The size of the GSCCs is shown in figure 1. In addition to seasonal variation in the size of the GSCCs, there are clear percolation-type transitions (Stauffer & Aharony 1992) characterized by a sudden increase in the size of the GSCCs as time-periods with more movements are considered. According to the distributions of strong component sizes (figure 3*a*, note the log–log scale), below the percolation threshold, the network of sheep movements are fragmented into many disconnected components of small size. Above the percolation threshold (figure 3*b*), a clear giant (largest) strongly connected component emerges with a size some 100 times greater than the next largest strong component.

The size of GSCCs in the SMNs represents a lower bound on the maximum number of nodes that a newly introduced infectious agent might reach. The upper bound is given by the size of the giant weakly connected component (GWCC; Schwartz *et al*. 2002). The GWCC contains the GSCC plus all the nodes that can connect to the GSCC in only one direction. During an epidemic started from nodes in the GSCC, only nodes that are destinations of directed connections starting in the GSCC could be infected apart from the nodes in the GSCC.

**Figure 4:**
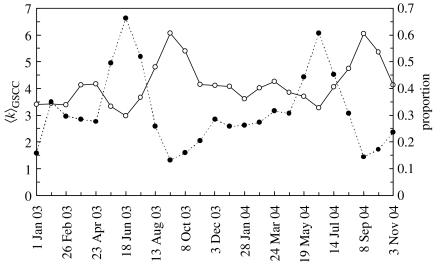
The average number of connections per node in the GSCCs (〈*k*〉_GSCC_) is shown by the continuous line (left-hand axis). The proportion of edges that join two different nodes in both directions is shown by the dotted line (right-hand axis).

Each GSCC was isolated from the containing network. The average number of links per node, 〈*k*〉_GSCC_, within the GSCCs is given in figure 4 (continuous line). The number of bidirectional links within the GSCCs (i.e. that run between the same nodes in both directions) is also presented as a proportion out of the total number of links in figure 4 (dotted line). The proportion of such links is inversely correlated with the average number of connections per node (〈*k*〉_GSCC_). A high proportion of bidirectional links limits the potential spread of an epidemic. The values of 〈*k*〉_GSCC_ within the GSCCs present the same seasonal variation as 〈*k*〉. Both the in and out degree distributions within the GSCCs show the same heterogeneity as seen in figure 2.

It is well known that in undirected networks (equivalent in a directed network to connecting two nodes by two directed links, one in each direction), the distribution of contacts determines how infectious disease may spread on a network, with a high variance promoting disease spread. For infinite, undirected scale-free networks with an infinite variance in the numbers of contacts, an epidemic can spread even for infinitesimally small transmission rates (May & Lloyd 2001; Pastor-Satorras & Vespignani 2001). However, in directed networks, the extent to which heterogeneity in the number of contacts aids disease spread depends on the correlation between the in and out degrees of nodes (Schwartz *et al*. 2002). The correlation between the in and out degrees of the nodes in the GSCCs were quantified using the Pearson product-moment correlation coefficient $\left(-1\leq r_{0}\leq 1\right)$. Results are summarized in table 1. The high positive correlation indicates the presence of nodes that are both likely to become infected and to transmit infection, facilitating disease transmission. The above correlation describes the behaviour of individual nodes and the covariance of the nodes' in and out degree plays a key role in determining the epidemic outbreak threshold in network based models (Diekmann & Heesterbeek 2000; Kao *et al*. 2006).

We now turn to the higher-order relationships between nodes. Most social networks show assortative mixing: highly connected nodes tend to link to other highly connected nodes and less well connected nodes to other poorly connected nodes (Newman 2002, 2003). By contrast, technological networks (e.g. WWW, Internet, transport networks) often show disassortative mixing, with highly connected nodes connecting to less well-connected nodes. Assortatively mixed networks are resilient to random and even targeted removal of nodes and the GSCC size is unaffected unless a significant proportion of highly connected nodes are removed (Newman 2002). Therefore, control in such networks is difficult unless precise and effective targeted control is used. Disassortatively mixed networks are less resilient to random and targeted removal, and therefore control is easier to implement. On disassortative networks, disease spread is at a disadvantage compared to the assortatively mixed case, especially for small transmission rates (Newman 2002). In undirected, infinite networks with an infinite variance in node degree, with or without degree correlations, epidemic outbreaks can happen even for infinitesimally small transmission rates (Boguna *et al*. 2003).

Newman (2003) proposed a measure of mixing for directed networks:

(3.1) $$r_{1}=\frac{\sum_{i}j_{i}k_{i}-M^{-1}\sum_{i}j_{i}\sum_{l}k_{l}}{\sqrt{\left[\sum_{i}j_{i}^{2}-M^{-1}{\left(\sum_{i}j_{i}\right)}^{2}\right]\left[\sum_{i}k_{i}^{2}-M^{-1}{\left(\sum_{i}k_{i}\right)}^{2}\right]}}.$$

Here, *j*_*i*_ and *k*_*i*_ are the ‘excess’ in degree and out degree of the nodes that the *i*th edge leads out of and into respectively, and *M* is the number of edges. The excess degree is the real degree of the node minus one, to account for the edge that is considered. The values of *r*_1_ range from [−1,0) for disassortative networks, and from (0,1] for assortative networks. For random networks with no degree correlation *r*_1_≈0. Values for *r*_1_ are presented in table 1 for the GSCCs. All are negative indicating disassortative mixing. This mirrors the typical trading pattern where direct movement between markets is illegal and highly connected markets typically trade with less well-connected farms. Frequent connections between highly and less well connected nodes slow the spread of the disease when compared to randomly or assortatively mixed networks.

A network is clustered if any two nodes *j* and *k* connected to a node *i* are in turn likely to be connected to each other. A high degree of clustering can reduce the extent of an epidemic (Eames & Keeling 2003) and can increase the efficacy of control measures such as contact tracing (Kiss *et al*. 2005). An upper estimate of the clustering coefficients within the GSCCs is computed by considering each directed link as being bidirectional. Soffer & Vasquez (2005) showed that the value of the classically defined clustering 〈*c*〉 (i.e. the average of the local clustering coefficient of each individual node (Albert & Barabási 2002)) can diverge from the value of *C* (i.e. the ratio of all possible triangles to all possible triples in the network), even when both are computed on the same network. The local clustering coefficient for an individual node *i* is defined as $c_{i}=E_{i}/(k_{i}(k_{i}-1)/2)$. Here, *k*_*i*_ is the number of nodes directly connected to node *i*. The value of *E*_*i*_ is the number of edges between the *k*_*i*_ neighbours of node *i* and $k_{i}(k_{i}-1)/2$ is the maximum number of potential edges among the neighbours of node *i* (Albert & Barabási 2002). In the $k_{i}(k_{i}-1)/2$ term, the degrees of the neighbours of node *i* are not considered and it is assumed that each neighbour can potentially have (*k*_*i*_−1) links connecting it to all the other remaining neighbours. If the total possible number of edges between neighbours is based on the degree of the neighbours the $k_{i}(k_{i}-1)/2$ expression changes and new clustering coefficients $\langle \tilde{c}\rangle$ and $\tilde{C}$ can be computed. This method reconciles the difference between the former two clustering coefficients and filters out degree correlations between connected nodes. Clustering coefficients were typically of order 0.01 (see table 1). Taking into account the directionality of the links, which is relevant to disease transmission, would further decrease the already small clustering coefficient. These low levels of clustering reflect the absence of market-to-market interactions (banned by legislation following the 2001 FMD epidemic; Bourn 2003), and relative rarity of farm-to-farm connections compared to farm-to-market and market-to-farm links. Higher-order clustering coefficients were also computed (i.e. ratio of connected loops of four to all connected quadruplets); however, all values were of the same order or smaller than those presented in table 1.

**Figure 5:**
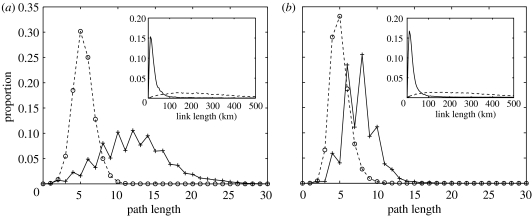
The distributions of path lengths for the SMNs starting on (*a*) 19 May 2004 and (*b*) 8 September 2004 (continuous lines). The dashed lines represent the path length distribution of random networks built by considering the same number of nodes and the same in and out degree for the nodes as in the SMNs. In the insets, the link length distributions for the SMNs (continuous lines) and for the equivalent random graphs (dashed lines) are plotted.

Next, we considered the average and distribution of the shortest path lengths between all possible pairs of nodes (figure 5) within two SMNs, starting on 19 May 2004 and 8 September 2004, respectively, providing contrasting scenarios of low and high levels of activity. These were compared with randomly connected networks, generated using the same nodes and degrees as in the SMNs but with *r*_1_≈0. In the SMNs, even-path length are more common; since most nodes represent farms, there is limited trading directly between farms and no trading directly between markets. This gives the networks an almost bipartite structure, in contrast with random network, where the distribution of path lengths is smoother.

The geographical location of the majority of the premises is known from census data. Based on the coordinates, for both SMNs and the corresponding randomly generated networks, the physical length of each link within the network was calculated where the coordinates of endpoints were known. The distribution of link lengths (figure 5, insets) for both periods is very similar. In the SMNs, there is much higher proportion of short-distance interactions than in the random networks. The average link length in the SMNs is considerably lower than that for the random networks (see table 2). This is consistent with the local network structure found by Kao *et al*. (2006). Though the average link length of the SMNs is considerably lower than for the randomly generated networks, the average path length of the SMNs was considerably closer to that for the corresponding random networks (see table 2), especially for the more densely connected SMN. Clustering in both SMNs and random networks is small, however, the geographically structured local interactions and the higher proportion of even path lengths are features unique to the SMNs that differentiate these networks from random ones. Although structurally the SMNs and random networks are different, the SMNs are well connected with their average path length being close to that computed using random networks. This shows that the SMNs present ‘small-world’ type features.

### 3.2 Epidemic simulations

Many theoretical network models assume random mixing with no correlation between the degrees of connected nodes. We refer below to these types of networks as random networks. As our networks are disassortative, we further investigate how mixing affects disease dynamics and spread by comparing epidemic simulations on a SMN with simulations on randomly connected networks with the same number of nodes and node degrees. For this purpose, the SMN starting on 8 September 2004 with 47 047 active nodes was chosen as a well-connected network on which disease can spread to a large proportion of the nodes. The size of GSCC in this SMN is 12 759 compared to an average GSCC size of 11 200 for the randomly rewired networks.

**Figure 6:**
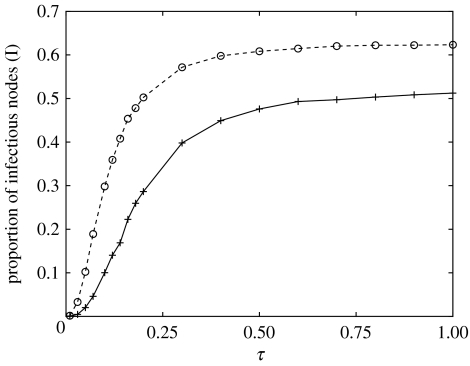
The average proportion of infectious nodes (I) at the end of a four-week epidemic for the SMN starting on 8 September 2004 (continuous line) and for the corresponding random graph (dashed line). The confidence intervals are smaller than the symbols.

The severity of disease was measured as the average proportion of infectious nodes (I) at the end of the four-week period. In figure 6, the average proportion of infectious nodes is plotted for both the SMN and the corresponding random networks versus the transmission rate *τ*. The number of infectious nodes is higher on random networks. Disassortative mixing within the SMN slows the epidemic spread. In the random networks, however, there are no correlations, and therefore as the epidemic progresses, it is more likely to find those nodes that have many connections. An important factor here is the interaction between the limited time for which the epidemic can spread (four weeks) and the contact network structure. A disease that spreads on a disassortative network needs a longer time to run its course and sample the network than the same disease spreading on a random network. Therefore, the average proportion of the infectious nodes measured in time-limited epidemics does not accurately reflect the natural long-term disease dynamics and contact network structure.

**Figure 7:**
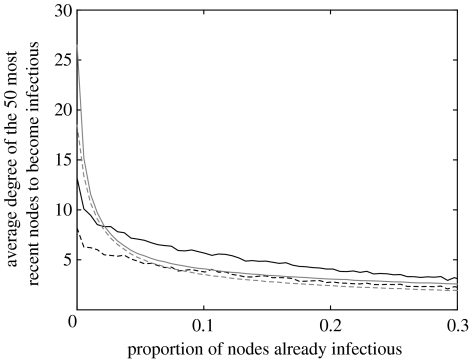
The average in (continuous line) and out (dashed line) degree of the *m* (=50) most recent nodes to become infectious for the SMN starting on 8 September 2004 (black lines) and for the corresponding random graph (grey lines) for *τ*=0.5. Different values of *m* produce similar qualitative behaviour.

Comparing the evolution of the average in and out degree of the *m*(=50) most recent nodes to become infectious during the epidemic on the SMN and random networks, figure 7 shows that on random networks the epidemic preferentially spreads to nodes with high in and out degree (Barthélemy *et al*. 2004). For the simulated epidemics on the SMN, the average in and out degree of new infectious nodes falls more slowly and presents less variation over time compared to the random network case, reflecting the connectivity pattern where infection alternates between highly and poorly connected nodes.

**Figure 8:**
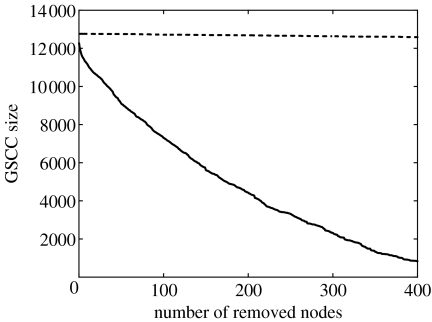
The effect of removing nodes according to their in times out degree (continuous line) on the GSCC size. For comparison, the dashed line represents the random removal of nodes. For both control methods, the starting network is the SMN starting on 8 September 2004.

In random networks, targeted removal of highly connected nodes is an effective epidemic control measure (Albert *et al*. 2000; Cohen *et al*. 2000; Newman 2002; Madar *et al*. 2004). Here, where the network is dissortative, targeting highly connected nodes may still be effective, as they may act as bottlenecks in the transmission process. The movement data allows identification of suitable control targets. We ranked nodes in the SMN starting on 8 September 2004 based on the product of the nodes' in and out degree. This product reflects the likelihood of the node of both becoming infected and transmitting infection. Out of the top 400 most highly connected nodes, all were unique market-days except seven show grounds, three farms and one veterinary premises. In figure 8, we compare targeted versus random removal of nodes by re-computing the size of the GSCC for both cases. The size of the GSCC is plotted against the number of removed nodes, showing that, as expected, targeted removal—highest-ranked nodes removed first—is much better at reducing the size of the GSCC and limiting the extent of a possible epidemic. Random removal has a less significant effect with only a small reduction in the size of the GSCC.

## 4. Discussion

In this analysis, we have reconstructed the networks of contacts within the GB sheep industry, based on livestock movement records. While there have been several recent analyses on detailed networks, the explicit characterization of GB livestock movement data is exceptional, particularly among epidemiologically relevant datasets.

The clear seasonality in the sheep trading pattern as highlighted by figures 1, 3 and 4, identifies periods of intense trading around August and September. Therefore, an epidemic that starts during this period has the potential to be widespread and reach many different parts of the livestock network. Thus, enhanced biosecurity and surveillance during this period is likely to benefit disease prevention and control. It is encouraging to note that most time of the year there is a reasonably low risk for a wide spread epidemic within the sheep industry. This most likely reflects policy changes implemented after the 2001 epidemic (Bourn 2003) as the widespread, rapid movement of older ewes in February was widely held to be the culprit behind the early characteristics of the epidemic (Kao 2002).

Small-world contact structures (Watts & Strogatz 1998) have previously been found in livestock contact networks in the GB. Webb (2005) investigated contact within the GB sheep industry based on geographical proximity and attendance at agricultural shows and found that a small number of long-range links was consistent with small-world effects. Christley *et al*. (2005) identified small-world network structures in the GB cattle movement network, with high heterogeneity in the number of contacts per node. The presence of a very small number of shortcuts in small-world type networks with highly localized structure ensures good connectivity between nodes and such networks are prone to disease spread. The comparison between the SMNs and randomly generated networks reveals a geographically local structure within the sheep industry. While the clustering in both the SMNs and random networks is small, there are important structural differences as shown by figure 5. Despite these, the average path length for the different networks is comparable (see table 2), especially for the periods of intense trading, and the SMNs are well connected. Thus, our analysis is indicative of a small-world type behaviour in the network of sheep trading in GB in the sense that the average length of possible paths between the nodes of the SMNs is small despite the clear structural differences when compared to random networks.

The comparison of SMNs to random networks conserved the degree distribution and the in and out degree of nodes. This allowed us to investigate the effect of the connectivity pattern (disassortative mixing) on disease spread by using an individual-based model to simulate epidemic spread on the two different networks. Targeting control (e.g. surveillance, tighter biosecurity measures) at highly connected nodes proves to be a very effective way of controlling disease (figure 8). The highly connected nodes are potential ‘super-spreaders’ (Hethcote & Yorke 1984) with many in and out connections and these nodes are therefore likely to become infected and to transmit the disease. Most of these nodes are markets and they require extra attention during periods of intense activity.

Assortativity and disassortativity in network connections represent a departure from proportionate mixing (Barbour 1978). Under proportionate mixing, the probability of connection from a node with *i* outward connections to a node with *j* inward connections is purely proportional to *i*×*j*. Non-random mixing can occur at a variety of levels, from preferential movement between particular premises types through local clustering, up to large-scale community structure. Identifying the network features responsible for the departure from proportionate mixing and their implications for disease dynamics is a key step when the efficacy of different epidemic prevention and control measures has to be evaluated.

While the livestock movement dataset is exceptional, the ability to electronically identify and record information is increasing and thus well-described real networks will inevitably become more common. Here, we have concentrated on the properties of static and unweighted directed networks corresponding to the livestock movements over fixed time-periods and identified key patterns in the sheep network that differentiate it from random networks. While both the well-known scale-free and small-world properties are relevant, the network shows clear seasonal changes in behaviour and unusual clustering and node correlation properties. Further analyses will consider the effect of the timing and weighting of movements and relate changes in the patterns of movements to potential changes in the effective transmission rates per movement. For example, the marked change in the proportion of bidirectional links over the year (see figure 4) may reflect a possible temporal variation in the types of trading of sheep over the year. These trading characteristics may alter the likelihood of transmission at different periods in the year, thereby changing the characteristics of the epidemiological network of truly infectious contacts (Kao *et al*. 2006) even if the social network of potentially infectious contacts is well known. Identifying how these two interpretations of network data differ will be a critical part to translating theoretical results into practical control measures.

## Figures and Tables

**Table 1:** The correlation between the in and out degree of nodes (*r*_0_), the mixing measure (*r*_1_) and clustering coefficients $\left(\langle c\rangle ,C,\langle \tilde{c}\rangle ,\tilde{C}\right)$ for the GSCCs obtained from SMNs containing four weeks worth of movements starting on the dates indicated.

	*r*_0_	*r*_1_	〈*c*〉	*C*	$\langle \tilde{c}\rangle$	$\tilde{C}$
21 May 2003	0.9167	−0.2956	0.0425	0.0047	0.0476	0.0438
18 Jun 2003	0.9810	−0.3266	0.0522	0.0017	0.0565	0.0488
16 Jul 2003	0.9480	−0.1252	0.0313	0.0023	0.0338	0.0251
13 Aug 2003	0.6237	−0.2368	0.0322	0.0042	0.0351	0.0312
10 Sep 2003	0.5690	−0.1318	0.0474	0.0031	0.0507	0.0492
8 Oct 2003	0.6976	−0.2543	0.0508	0.0041	0.0544	0.0472
5 Nov 2003	0.9167	−0.1296	0.0571	0.0081	0.0633	0.0495
21 Apr 2004	0.8068	−0.2768	0.0078	0.0011	0.0083	0.0055
19 May 2004	0.8936	−0.2257	0.0316	0.0063	0.0361	0.0451
16 Jun 2004	0.9499	−0.2455	0.0359	0.0024	0.0400	0.0530
14 Jul 2004	0.9101	−0.1166	0.0431	0.0047	0.0473	0.0454
11 Aug 2004	0.7135	−0.2443	0.0404	0.0047	0.0439	0.0356
8 Sep 2004	0.6005	−0.1039	0.0337	0.0015	0.0362	0.0275
6 Oct 2004	0.7089	−0.2884	0.0410	0.0027	0.0438	0.0310
3 Nov 2004	0.8379	−0.2159	0.0356	0.0019	0.0389	0.0252

**Table 2:** Sheep movement network characteristics for contrasting scenarios of low and high levels of activity compared to the corresponding randomly rewired networks.

	19 May 2004		8 Sep 2004	
		
	SMN	rewired network	SMN	rewired network
average link length (km)	47	265	41	267
average path length	12.3	5.4	7.7	5.1
diameter	36	14	24	19
